# The advancement of nanosystems for drug delivery in the prevention and treatment of dental caries

**DOI:** 10.3389/fcimb.2025.1546816

**Published:** 2025-02-11

**Authors:** Han Du, Zheng Wang, Shenglan Long, Yiding Li, Deqin Yang

**Affiliations:** ^1^ Stomatological Hospital of Chongqing Medical University, Chongqing Key Laboratory of Oral Diseases, Chongqing, China; ^2^ Department of Conservative Dentistry and Endodontics, Shanghai Stomatological Hospital & School of Stomatology, Fudan University, Shanghai, China; ^3^ Shanghai Key Laboratory of Craniomaxillofacial Development and Diseases, Fudan University, Shanghai, China

**Keywords:** dental caries, biofilm, nanosystems for drug delivery, remineralization, antibacterial agents

## Abstract

The dental caries remains a globally prevalent disease. Although its incidence has decrease due to enhancements in sanitation policies and public health measures, the treatment and prevention of dental caries still pose significant challenges. Within the oral cavity, traditional drug delivery systems suffer from limitation such as inadequate tissue penetration, short duration of action at target site, and low specificity, which minimally affect the prevention and treatment of dental caries. Consequently, nanosystem for drug delivery, offering enhanced drug stability, solubility, and bio-availability while reducing side effects, garnering attention increasing attention in the fight against dental caries. Therefore, this review examines the role of nanosystems for drug delivery in combating dental caries by inhibiting bacteria survival, biofilm formation, demineralization, and promoting remineralization, and exploring their potential to become the mainstream means of prevention and treatment of dental caries in future.

## 1 Introduction

Dental caries, primarily caused by bacterial infections, is a progressive and destructive disease affecting the hard tissue of teeth. When dental caries develops into periapical inflammation, it can not only lead to severe pain for the patient but also cause potentially sepsis due to the spread of localized infection (Pitts et al., 2017). Statistical data suggests that dental caries is one of the most prevalent diseases worldwide, “A Systematic Analysis for the Global Burden of Disease 2017 Study” reveals that the out of 3.5 billion cases of oral diseases, and including 2.3 billion cases of untreated permanent tooth decay (GBD 2015 Disease and Injury Incidence and Prevalence Collaborators, 2016; Bernabe et al., 2020).

The formation of dental caries initiates when salivary proteins adhere to the tooth surface to form an acquired pellicle, which is colonized by bacteria quickly, eventually leading to form the plaque biofilm (Bowen et al., 2018). The plaque biofilm comprises various cariogenic bacteria, particularly *Streptococcus mutans, lactobacillus*, and *actinomycetes.* These bacteria metabolize carbohydrates (primarily free sugars) in the dental biofilm, producing acids through glycolysis (Zhang et al., 2022), thereby reducing the oral pH value. With the continuous acid and the lower pH value of oral environment, which leads to the loss of minerals such as calcium and phosphate from the enamel into the external environment. The gradual loss of mineral particles in hydroxyapatite results in demineralization, thus forming carious lesions (Selwitz et al., 2007). Due to the unique physiological and anatomical characteristics of the tooth (Marsh, 2010) and the growth of maturing cariogenic bacteria is protected by Extracellular polysaccharides (EPS) (Sims et al., 2018), effectively removing the plaque biofilm and the expected performance of the local antimicrobial drug pose significantly challenges.

**Figure 1:**
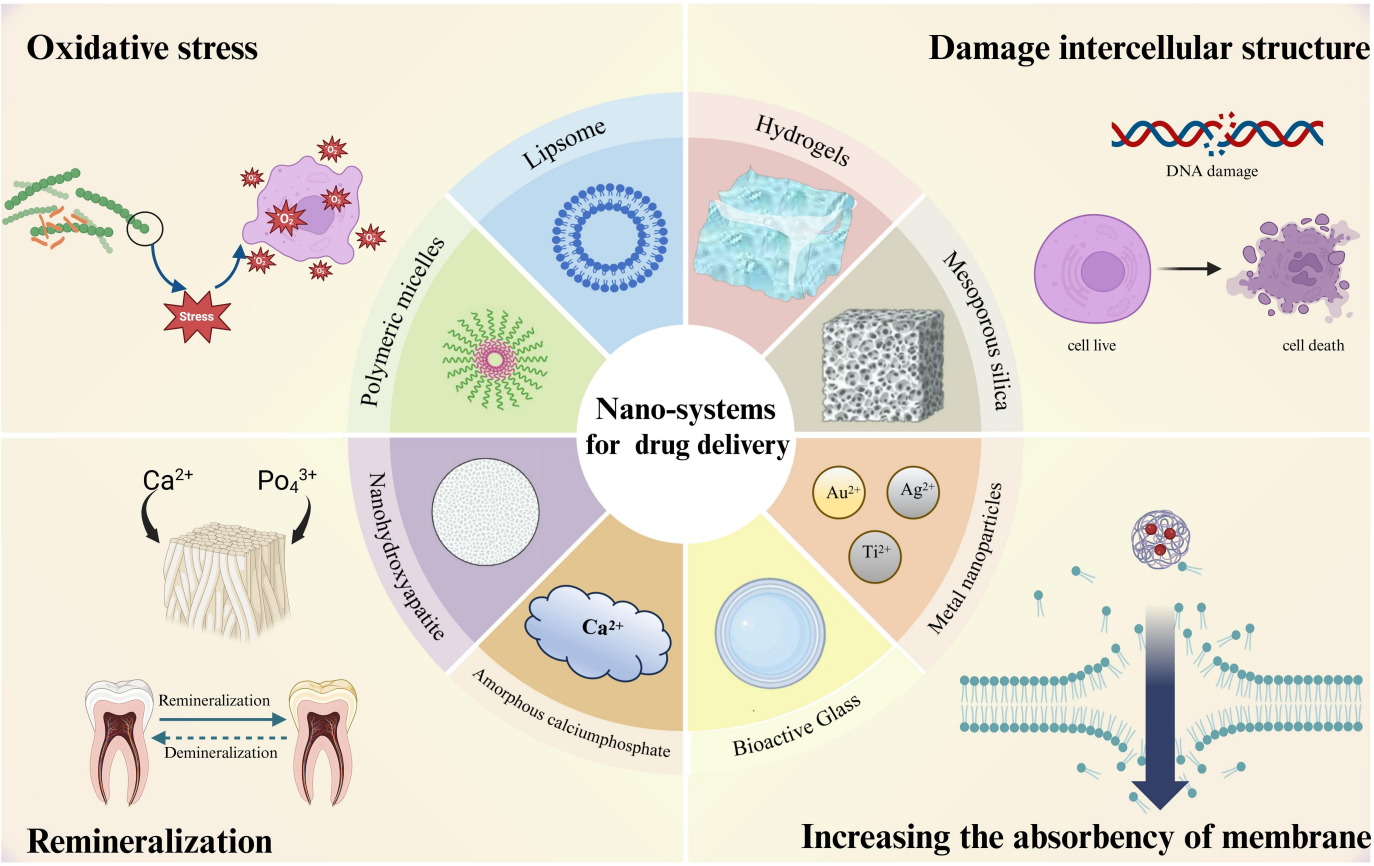
Nanodelivery systems currently used for anti - caries and their anti - caries mechanisms.

Nowadays, most traditional anti-caries drugs exhibit low bioavailability and poor solubility, leading to they are eliminated from body quickly. Furthermore, the emergence of drug-resistant pathogens and dose-dependent adverse effects of certain chemicals severely limit the efficacy of traditional therapies (Renugalakshmi et al., 2011). Consequently, anti-caries nanosystems for drug delivery have become one of the breakthrough points in the research of caries prevention and treatment. Nanosystems for drug delivery utilize the nanoparticles with a range from 1 to 100nm (Murthy, 2007), as carriers to encapsulate, safeguard, and convey drug molecules to targeted bodily regions. Research have indicated that the nanoparticles hold remarkable potential in drug delivery (Khizar et al., 2023), attracting much attention by improving the drug stability, solubility, and bioavailability while mitigating side effects, thus enhancing the therapeutic effectiveness (Qiao et al., 2022) The most common drug delivery nanoparticles include liposomes, solid lipid nanoparticles, inorganic nanoparticles (nanosilica, gold, silver), polymer nanoparticles, and polymeric micelles (Torchilin, 2014). These nanoparticles play a key role in drug delivery. Compared with traditional drug delivery systems, nanosystems for drug delivery offer superior performance in suppressing cariogenic bacteria and advancing dental remineralization. This review elucidates the anti-caries effectiveness of nanodelivery systems, spotlighting their role in curbing the growth of cariogenic bacteria, biofilm development, and demineralization, and fostering remineralization, alongside their prospective utility in forthcoming anti-caries interventions (**Figure 1**).

## 2 Anti-biofilm

### 2.1 Liposomes

Liposomes are spherical nanovesicles composed of phospholipids and cholesterol, which carry a wide range of diagnostic or therapeutic hydrophobic and hydrophilic medications. They can also deliver and protect encapsulated compounds from the effects of metabolic processes (Liu et al., 2021). Studies have shown that liposomes can adsorb onto hydroxyapatite, which enables them to adhere the enamel surface for extended periods (Nguyen et al., 2010), prolonging their presence in the oral cavity. This adsorption capability, coupled with their capacity to encapsulate lipophilic or hydrophilic drugs, contributes to their effective antibacterial action against the plaque biofilm. Moreover, by physically covering the enamel surface, liposomes offer additional protection to the enamel (Nguyen et al., 2011).

Currently, the efficacy of medicine for the early prevention and treatment of dental caries is impacted by the diversity of the oral microbiome, mainly due to the drugs, poor biofilm-targeting capabilities. Lactoferrin has been proven to inhibit the proliferation of *S. mutans* and the ability of oral pathogens to form biofilms. P Habibi et al. used the thin-layer distribution method to prepare lactoferrin-containing nano-liposomes to evaluate their effect on the biofilm formed by *S. mutans*. The results showed that nano-lactoferrin was more effective in reducing the colony-forming units (CFU) of the *S. mutans* biofilm than free lactoferrin, and nano-liposomes of lactoferrin also significantly reduced the lactate production by *S. mutans* (Habibi et al., 2022). Similarly, research has shown that lipopolysaccharide -encapsulated magnolol (MAG) and fluconazole (FLC) to address their hydrophobicity and rapidly release the drugs in a pH-sensitive manner (Luo et al., 2023). This drug delivery system successfully overcame the hydrophobic characterization of the drugs, enhancing their antimicrobial efficacy against *C. albicans* and *S. mutans*. Furthermore, due to the modification of these composite particles with pyrophosphate ions (PPI), which exhibit good affinity with hydroxyapatite, the PPI - Mag/FLC-LPs can deliver drugs to teeth with high affinity, presenting a novel perspective on the use of nanosystems for drug delivery-based for cooperative drug delivery in oral anti-biofilm treatments.

The expression levels of histatin-1 in populations across different age groups who are prone to dental caries are significantly lower than in those without caries, suggesting that histatin-1 may have important implications for the prevention and treatment of dental caries (Wang et al., 2018a, 2018b). With high affinity for enamel surfaces, activities in the formation of acquired enamel pellicle and the N-terminal domain of histatin-1 could competitively reduce the adhesion of *S. mutans* onto HAP surfaces, Zhang and coworkers synthesized a novel biomimetic peptide DK5 (DpSHEK) inspired by histatin-1, it could adsorb to the surface of acid eroded HAP and guide the nucleation of calcium and phosphorus for enamel remineralization. Although it has potential for remineralization of initial enamel, it cannot be used clinically because of its remaining problems including quick elimination, easy dilution and degradation in the oral cavity.

Consequently, they prepared histatin-1 derived peptide-loaded liposomal system (DK5-Lips) indicated that DK5-Lips exhibited a sustained release profile, excellent stability in saliva, DK5-Lips group had higher surface microhardness recovery, shallower caries depth and less mineral loss in bovine enamel, moreover, it has no significant toxicity on human gingival fibroblasts (HGFs) (Zhang et al., 2023). The novel liposomal delivery system for a novel peptide derived from (DK5-Lips) as a new biomimetic mineralization strategy against initial enamel caries, the system could exert significant anti-caries effect both *in vitro* and *in vivo*, evidenced by the increased level of remineralization and the reduced degree of caries decay.

Xiao et al. developed a liposome-encased formulation of indocyanine green (ICG) and rapamycin for drug delivery (ICG-rapamycin). Their investigation revealed that ICG-rapamycin, when subjected to 808 nm laser excitation, significantly enhances the levels of reactive oxygen species (ROS) and temperature, thereby activating photodynamic and photothermal mechanisms to elicit antibacterial effects. Furthermore, ICG-rapamycin promotes bacterial motility by elevating intracellular ATP concentrations within bacterial cells and simultaneously inhibiting both bacterial adhesion and biofilm formation. This innovative anti-biofilm strategy effectively addresses the challenge of disease recurrence resulting from the proliferation of residual viable bacteria, which can generate biofilms post-antibacterial treatment. Additionally, near-infrared (NIR) laser excitation facilitates M2 polarization and augments TGF-β concentrations, leading to a reduction in cellular inflammatory responses and improving the phagocytic activity of macrophages toward bacteria. The results of this investigation indicate that ICG-rapamycin has the potential to effectively treat and prevent common biofilm-associated oral diseases by modulating the microbial-cellular microenvironment, thereby offering considerable promise for future applications in dental clinics (Xiao et al., 2023).

Curcumin, functioning as a natural exogenous photosensitizer, interacts with ground-state molecular oxygen to produce reactive oxygen species (ROS) under blue light excitation (Luby et al., 2019). The recent study conducted by Hu and coworkers designed a liposome with adhesion properties to deliver curcumin (Cur@LP) into the biofilm. Curcumin can be released from the liposome near the biofilm and exert an antibacterial effect by dispersing the biofilm under blue light irradiation. The result indicates that Cur@LP group had the least residual *S. mutans* biofilm when compared with other groups. It proves that the Cur@LP has better activity of antibacterial and adhesion onto the *S. mutans* biofilm but no significant cytotoxicity compared with curcumin group. Clinically, the convenience offered by the widespread application of the blue light source and the adhesion ability to *S. mutans* biofilm will make the Cur@LP a broad application prospect (Hu et al., 2023). Liposomes show promise for targeted drug delivery in oral care, offering enhanced biofilm control, remineralization. However, more clinical experiments are still needed to prove its safety for its application.

### 2.2 Poly - (lactic-co-glycolic acid) (PLGA)

The combination of Lactic Acid (LA) and Glycolic Acid (GA) creates a copolymer system called PLGA, PLGA nanoparticles are used for sustainable drug delivery systems because of their excellent bioavailability, biocompatibility, biodegradability, small size, and ability to release medicine over time (Vlachopoulos et al., 2022; Narmani et al., 2023). One of the main uses of PLGA is in delivering drugs, and it can be shaped into large-scale structures (like scaffolds or gels), microparticles (MP), or even smaller nanoparticles (NP). In the realm of healthcare, these PLGA formats have shown their potential to encapsulate various kinds of medicinal agents, including antibiotic, drugs that reduce inflammation, and antioxidants, to fulfill therapeutic objectives. Crucially, materials based on PLGA can shield the encapsulated drugs from degradation early and ensure a steady release of medication, making them exceptionally well-suited for treatments that span a longer duration.

Chlorhexidine (CHX) has been widely used in dentistry, usually in the form of an oral rinse, to prevent the formation of dental plaque and calculus, as well as to treat oral inflammation. CHX has been considered the “gold standard” (Hetrick et al., 2009) among antimicrobial agents due to its broad-spectrum antimicrobial effects. A single direct pre-treatment with CHX on the acid-etched dentin matrix significantly enhances the degradation resistance of the resin-dentin bond interface. However, without a drug-release source, the concentration of CHX cannot be guaranteed, and the non-biodegradable CHX-modified bonding resin could hinder the release of CHX due to the thickness of the adhesive layer, adversely affecting the dentin bonding system. Therefore, Priyadarshini et al. loaded CHX onto PLGA nanoparticles. These CHX@PLGA nanoparticles exhibited low cytotoxicity and strong antibacterial abilities, and CHX was continuously released over 28 days (Priyadarshini et al., 2017). In addition, when CHX@PLGA nanoparticles were in aqueous solution, they could penetrate up to 10 μm deep into dentinal tubules and tightly bond with the resin after penetration of the adhesive. It demonstrated the potential application of CHX@PLGA entering dentinal tubules for adhesive restorative treatments and its capability to deliver other drugs already used in dental treatments, thereby expanding the therapeutic methods for oral diseases continuously.

Additionally, F.O. Sousa et al. discovered that PLGA composites containing chlorhexidine diacetate and Digluconate demonstrate potent antimicrobial properties against *S. mutans* (Francisco Fábio Oliveira de et al., 2021). Specifically, the digluconate encapsulated in a solid form within PLGA exhibits a rapid release. Moreover, diacetate-loaded PLGA particles ensure a consistent and sustained release of the drug over a period of 120 days. Advances in nanotechnology and targeted delivery methods may enhance the capabilities of PLGA-based systems, allowing for improved patient outcomes and the development of personalized strategies for oral health maintenance and caries prevention.

### 2.3 Mesoporous silica nanoparticles (MSN)

Mesoporous materials refer to nanomaterials with pore sizes in the range of 2 to 50 nm, among which mesoporous silica has attracted considerable attention due to its excellent controllable drug delivery capabilities (María et al., 2022; Bianca et al., 2023). Mesoporous silica nanoparticles (MSNs) maintain a certain chemical stability, surface functionality (high surface area and adjustable pore size), biocompatibility, and biodegradability because of their unique mesoporous structure. With these properties, MSNs ensure the controlled release and targeted delivery of various drug molecules (Komal Sadashiv, 2015; Lanlan et al., 2023). Moreover, MSNs can respond to certain stimuli during the loading, protection, and transport of drugs to release them. Therefore, MSNs can load antimicrobial drugs and incorporate them into resin composites to produce an anticariogenic effect (Montserrat and María, 2020). In summary, due to their large surface area and pore size, which enable a higher drug load capacity and targeted drug delivery, MSNs enhance therapeutic efficacy while reducing side effects, exhibiting safer and more effective treatment outcomes.

Research has proven that L-arginine can be metabolized by the arginine deiminase system in oral bacteria, thereby raising the pH value of dental plaque and reducing the risk of caries (Marcelle et al., 2013). Researchers prepared MSNs loaded with L-arginine and integrated them into the dentin adhesive system (Arg@MSN@DAdh) (Marta et al., 2022). The experiments revealed that the adhesive system containing Arg@MSN@DAdh significantly reduced the populations of *S. mutans* and *Lactobacillus casei*, with no observed compromise in its physical, mechanical, and adhesive properties. Furthermore, this composite could continuously release L-arginine, exhibiting enduring antibacterial capabilities and preventing biofilm formation.

Studies have found that encapsulating CHX in MSNs enables it to penetrate the biofilms of *S. mutans* and interact with other microbes (Ya et al., 2011; Li et al., 2016); it even retains its biofilm-inhibitory effect after 50 hours. Based on this, Lu et al. developed biodegradable disulfide-bridged MSN, it could deliver silver nanoparticles and CHX (Ag-MSNs@CHX) concurrently (Lu et al., 2018). Experiments have shown that, due to its redox and pH-responsive release properties, this composite significantly inhibits the growth and biofilm formation of *S. mutans*, and its antibacterial effect is superior to that of an equivalent amount of free CHX. More importantly, no abnormal reactions were observed in mice after oral administration of Ag-MSNs@CHX, and Ag-MSNs@CHX significantly reduced the cytotoxicity of CHX to the oral mucosal epithelial cells.

Two-component signal transduction systems (TCSTS) are capable of modulating gene expression in response to external environmental changes. The VicRK system, one of the TCSTS, consists of a membrane-bound sensor (VicK) and a cytoplasmic response regulator (VicR). VicR is an essential regulator in exopolysaccharide (EPS) production which is one of the main cariogenic factors of S. mutans. It is reported that an Antisense vicR RNA (ASvicR) could bind to vicR mRNA, hindering the transcription and translation of the vicR gene. Moreover, ASvicR can inhibit EPS synthesis, bacterial growth, and cariogenicity of S. mutans, suggesting its potential as a strategy for caries prevention (Senadheera et al., 2005; Dongsheng et al., 2007; Lei et al., 2019; Yuting et al., 2023). Tian and coworkers had constructed a recombinant plasmid containing the ASvicR sequence (plasmid-ASvicR) and proved that it could reduce EPS synthesis, biofilm formation, and cariogenicity. However, the recombinant plasmids need protection from enzymatic degradation and require higher transformation efficiency. Consequently, they further synthesized and characterized aminated dendritic mesoporous silica nanoparticles (DMSNs-NH2) and demonstrated its capability to transport and maintain the integrity of plasmid-ASvicR (DMSNs-NH2-ASvicR). The result indicated that DMSNs-NH2 could effectively protect most of the plasmid-ASvicR from being degraded by DNase I. When loaded onto DMSNs-NH2, the plasmid-ASvicR was able to enter *S. mutans* suppress the expression of the vicR gene, which in turn reduced the synthesis of EPS and the formation of biofilms in S. mutans. Furthermore, cytotoxicity experiments revealed that DMSNs-NH2-ASvicR exhibited no cytotoxic effects, and Keys scores outcomes demonstrated that DMSNs-NH2-ASvicR significantly lowered caries incidence in rats. This suggested that DMSNs-NH2 can protect the plasmid-ASvicR against degradation effectively and enhance its penetration into the bacteria within the rat’s oral cavity. Demonstrating excellent biocompatibility, DMSNs-NH2-ASvicR sets a solid groundwork for future biomedical applications (Yuting et al., 2022).

In dental applications, MSNs demonstrate substantial antibacterial effects, effectively loading and releasing antimicrobial agents to reduce the risk of caries. Moving forward, ongoing research into the modification and functionalization of MSNs will further enhance their application potential in the prevention and treatment of oral biofilm-related diseases. Additional clinical studies will be essential to validate the long-term efficacy and safety of MSNs in biomedical applications.

### 2.4 Poly (amino amine) (PAMAM) dendrimers

PAMAM dendrimers, often referred to as “artificial proteins” due to their protein-like structure, are highly branched nanopolymers featuring both internal cavities and external terminal groups. These internal cavities facilitate the transport of drugs or ions (Qichao and Janet, 2015), while the external groups can be modified with various functional groups to act as carriers for drug delivery, thereby controlling the release of drugs. Notably, they exhibit both antibacterial and remineralization properties, making them effective in both treating and preventing dental caries. In aqueous solutions, PAMAM dendrimers tend to self-assemble into hierarchical structures, namely nanospheres, nanochains, microfibers, and eventually macroscopic aggregates (Jiaojiao et al., 2013). This self-assembly and the resultant hierarchical structures are capable of mimicking the function of amelogenin, which is crucial in the formation of dental enamel (Mirali and Thomas, 2021). Consequently, PAMAM dendrimers are considered a promising material for dental repair.

Previous studies showed that honokiol could inhibit the growth of many cariogenic pathogens, and could reduce acid production by cariogenic bacteria (Jun et al., 2004). However, its hydrophobicity limited its further application. Tao et al. encapsulated water-insoluble honokiol within the hydrophobic interstitial cavity of PAMAM, thereby endowing PAMAM with enduring antibacterial properties. They successfully prepared and characterized honokiol-loaded carboxyl-terminated PAMAM (PAMH), PAMH demonstrated low cytotoxicity, with its drug release dynamics elucidated via computational simulation analysis. At a low pH of 5.5, resulting in a relatively slow swelling rate and consequently a more gradual release of drug molecules in an acidic milieu compared to a neutral environment. The effective release of honokiol contributed to the sustained antibacterial properties of PAMH, They concluded that PAMH possesses antibiofilm-forming properties by inhibiting the proliferation of planktonic bacteria. Furthermore, PAMH facilitated enamel remineralization after pH cycling treatment *in vitro*, and animal studies supported its effectiveness in addressing carious lesions in rats (Tao et al., 2021).

Existing materials to induce dentin remineralization lack the ability to stabilize dentinal collagen which is the basic support for the growth of inorganic minerals and plays a role of mechanical support (Leo et al., 1999; Erika Kiyoko et al., 2020). The repeated low pH stimulation activates matrix metalloproteinases (MMPs) under pathological conditions such as dental caries, it could destroy the structure of dentinal collagen which leads to the induction of biomimetic remineralization losing its structural basis. Tao and colleagues constructed galardin-loaded poly (amido amine) (PAMAM)-NGV (PAMAM-NGV@galardin, PNG) to simultaneously induce collagen stabilization and dentin biomimetic remineralization.

In order to combat early dentin caries, NGV peptides and galardin demonstrated a dual collagen-protective effect, which lays the foundation for the dentin remineralization effect facilitated by PAMAM (Tao et al., 2022). The results suggested that in acidic environments, galardin can be more sustainably released, and a longer inhibitory effect on MMPs is achieved. The NGV peptides, modified on the surface of the dendrimer core, could form small clusters exhibiting synergistic movement over short ranges. These clusters could then create domain areas with varied properties on the PAMAM core’s surface, effectively restricting collagen movement. This restriction was beneficial for collagen crosslinking. PNG induced dentin remineralization in a collagenase environment *in vitro*, and animal experiments also indicated that PNG could effectively combat dentin caries in rats. PNG showed great potential for dentin repair in future clinical applications.

Fan et al. utilized PAMAM dendrimers with different terminal groups to treat artificially demineralized bovine incisors, aiming to quantify the remineralization effects of these PAMAM dendrimers in the subsurface demineralized enamel (Menglin et al., 2020). They employed Transverse Microradiography (TMR) and cross-sectional microhardness testing for the first time to evaluate the differences in remineralization capabilities of PAMAM dendrimers with different terminal groups. After treatment with PAMAM dendrimers possessing various terminal groups, the demineralized bovine teeth examined through TMR showed a significant reduction in the translucent zone. This reduction in the area indicates the deposition of minerals in the subsurface enamel, thereby achieving remineralization. Moreover, through Scanning Electron Microscope (SEM) analysis of the bovine teeth samples, they found that compared to the control group, the PAMAM dendrimer-treated group exhibited shallower lesion depths and less mineral loss. At the same time, these samples showed a more significant recovery in microhardness on the vertical cross-sectional surfaces and absorbed more mineral deposits. These results indicate that although enamel itself can remineralize to some extent in artificial saliva, the remineralization effect of PAMAM dendrimers is more pronounced. In addition, PAMAM-NH_2_ showed the strongest remineralization capability, followed by PAMAM-COOH, while PAMAM-OH had the weakest effect. The study also found that PAMAM-NH_2_ dendrimers could strongly adsorb to the enamel surface and form a stable bond with it. Therefore, PAMAM dendrimers with specific terminal groups exhibit strong enamel protection and remineralization potential, making them powerful candidates for the prevention and treatment of caries.

### 2.5 Chitosan

Chitosan is a natural polymer derived from the deacetylation of chitin, primarily sourced from the exoskeletons of marine crustaceans such as crabs and shrimp (Kamel et al., 2021). Its deployment as a drug delivery system, as well as its application in tissue engineering and as a therapeutic agent, has been intensively researched. Chitosan’s notable antibacterial, remineralization capabilities, and adherent properties to dentin, alongside its excellent biodegradability, biocompatibility, and non-toxicity, have made it an area of significant interest in caries prevention and treatment (Shelyn Akari et al., 2022). Under acidic conditions, chitosan captures hydrogen ions through its amino groups, thereby becoming positively charged. Through electrostatic forces, the molecule adheres to negatively charged surfaces, such as tooth enamel, making it a nano drug delivery carrier that can transport ions or active substances to the tooth surface (Degli Esposti et al., 2020).

Current preventive strategies, primarily centered on fluoride, can inhibit enamel demineralization to a certain degree; however, they do not effectively prevent persistent biofilm formation and may induce adverse effects such as fluorosis or alterations in oral and gut microbiota. Consequently, there is a pressing need for a safe and effective approach to preventing dental caries (Li et al., 2019). Jiang et al. successfully synthesized a novel type of nanoparticle (CSP NPs) that exhibits both colony-suppressing and enamel demineralization-inhibiting properties (Jiang et al., 2024). These nanoparticles demonstrated efficacy in eradicating a specific strain of *S. mutans* biofilms in the absence of antibiotics. Moreover, the nanoparticles were applied to the enamel surface, enabling the binding of calcium to impede demineralization. Results from animal studies and oral microbiome analyses indicated that the CSP NPs effectively prevented dental caries without adversely affecting the microbiota or host tissues. Moreover, curcumin within chitosan nanoparticles (CSNP-Cur) were encapsulated to assess their efficacy in disrupting biofilms formed by *C. albicans* and *Staphylococcus aureus* (Ma et al., 2020). They transferred microbial cultures onto medical-grade silicone sheets to initiate biofilm formation, then treating with CSNP-Cur. The structure of biofilm on the medical silicon and the viability of bacteria within biofilm were observed by Scanning Electron Microscopy (SEM) and Confocal Laser Scanning Microscopy (CLSM). The observations from SEM and CLSM showed that CSNP -Cur could effectively reduce the thickness of the biofilms and kill the microbes embedded in the biofilms on the silicone surface. In a similar vein, Jasem et al. found that amoxicillin-loaded chitosan nanoparticles demonstrated enhanced antibacterial properties compared to chitosan alone (Abdullah and Maha Abdul Kareem, 2023). Given chitosan’s versatility in forming drug delivery systems, such as gels, tablets, and micro/nanoparticles (Sevda et al., 2019), it exhibited a significant potential in preventing bacterial or plaque biofilm formation and demonstrated bactericidal properties, suggesting its utility as a prophylactic agent in early dental caries prevention.

Previous study had reported that the Cinnamaldehyde has anti-inflammatory and broad-spectrum antibacterial effects. It could be a potential anti-caries drug due to its strong effect of inhibiting *S. mutans* (Spartak, 2020; Harrison et al., 2021; Jishuai et al., 2022). Mu et al. design a novel nanosystem by loading Cinnamaldehyde (CA) into chitosan-based nanocapsules (CA@CS NC). The result indicated that CA@CS NC down-regulated QS gene, inhibited bacterial population effects such as biofilm formation and acid production, and better exerted the antibacterial effect of low-concentration CA. The keyes’ score showed the development of dental caries was inhibited in CA@CS NC group. Moreover, with an oil-based core and a positively potential CS shell, which are able to adsorb *S. mutans* through electrostatic interactions and slowly release CA (Ran et al., 2023).

There are existing studies that have proven that there are inhibitory effects of Chitosan on *S. mutans* and *Porphyromonas gingivalis*, and oral care products containing Chitosan (such as water-soluble Chitosan mouthwash and chewing gum) have been shown to inhibit plaque growth and bacterial proliferation (Chih-Yu and Ying-Chien, 2012; Zahra et al., 2020). When Chitosan is functionally modified with natural compounds, metal antimicrobial particles, and antimicrobial drugs, it exhibits enhanced antibacterial effects. The antibacterial action of Chitosan primarily stems from the interaction between the cationic Chitosan molecules and the negatively charged cell membranes, leading to abnormal cell permeability and changes, thus resulting in cell death and the leakage of cellular contents. Chitosan nanoparticles (NPs) have a relatively larger surface area, leading to stronger drug loading capabilities (Somaye et al., 2021; Ashwini and Arpana, 2015). In summary, Chitosan, as a nanocarrier for dental caries prevention, not only possesses antibacterial capabilities but also, when it serves as a drug carrier, enhances the antibacterial efficacy of the drug-Chitosan composite.

### 2.6 Polymeric micelles

Micelles are self-assembled from amphiphilic polymers at the critical micelle concentration (CMC). These self-assembled amphiphilic polymers, containing both hydrophobic and hydrophilic ends, are known as Polymeric micelles (Suguna et al., 2022), enabling drugs with low solubility to dissolve in Polymeric micelles (Yinglan et al., 2018). Polymer micelles have numerous advantages, such as targeted delivery, stable storage of drugs, due to their nanoscale size and narrow distribution characteristics, can protect drugs from oxidation. Polymer micelles can encapsulate poorly soluble small molecule drugs, enhancing efficacy while reducing toxicity. In preclinical animal models, polymer micelles have demonstrated improved pharmacokinetic properties and better safety (Duhyeong et al., 2020). Numerous natural products and their derivatives inhibit *S. mutans*, notably components found in orange and lemon essential oils. Such components, including phellandrene, enhance the proton permeability of the bacterial cell membrane and reduce the glycolytic activity of *S. mutans* in dental plaque (Jeon et al., 2011). Consequently, a novel polymeric micelle by conjugating Farnesal (Far) was developed to the amino groups of PEG, rendering it sensitive to low pH environments (Yi et al., 2020). This design aimed to selectively release Far in the oral environment prone to caries. They modified the polymeric micelles by coupling with pyrophosphate ions (PPi) and loading them with Far to enhance its solubility significantly, thus improving bioavailability and creating a new drug delivery system, PPi-Far-PMs. In a rat caries model, PPi-Far-PMs demonstrated a significant reduction in *S. mutans* count and continuous inhibition of its growth *in vivo* compared to the control group. Moreover, PPi-Far-PMs exhibited rapid binding to hydroxyapatite, facilitating Far release, enhancing retention in the oral cavity, prolonging the drug’s action, and providing a more potent anti-caries effect. PPi-Far-PMs proved more effective than free phellandral, demonstrating their potential for targeted antimicrobial therapy for caries and the delivery of other oral disease therapeutics. Polymeric micelles, serving as carriers for drugs with low water solubility, present extensive potential in oral healthcare. Xu and coworkers developed a novel stimuli-responsive multi-drug delivery system (PMs@NaF-SAP). The system utilized the acidic pH associated with tooth decay as a trigger. It featured MAL-modified PEG-b-PLL/PBA-sheddable micelles as nanocarriers, which are loaded with the antibacterial drug tannic acid (TA) and the restorative drug NaF. Additionally, the bioinspired salivary-acquired peptide DpSpSEEK (SAP) is attached to the micellar nanoparticles, ensuring specific adhesion to the tooth. This adhesion strength, combined with the pH-sensitive boronate ester linking TA and PBA, allows PMs@NaF-SAP to firmly attach to the tooth surface. It withstands the buffering action of saliva in the oral cavity, promoting the accelerated release of TA and NaF directly to the sites of caries as the oral microenvironment becomes more acidic. Both *in vitro* and *in vivo* measurements have confirmed the intelligent drug-released system exerts effective antibacterial adhesion and cariogenic biofilm resistance, inhibits enamel demineralization and promotes remineralization to prevent tooth decay and promote enamel restoration and they evaluated the safety and biocompatibility of PMs@NaF-SAP through cell viability assays. Using CCK-8 analysis, it was observed that at dilutions ranging from 1:20 to 1:100, cell viability exceeded 80%. PMs@NaF-SAP demonstrated significantly lower cytotoxicity compared to CHX. Cytoskeleton staining confirmed that, within this dilution range, the cells maintained healthy morphology and proliferation was unaffected. Consistent results were obtained via fluorescence quantitative analysis. Therefore, it demonstrates significant promise in broadening the limited clinical options currently available for the prevention of caries and the restoration of dental defects (Xu et al., 2023). By integrating bioactive natural compounds and targeted delivery mechanisms, polymeric micelles may revolutionize oral disease management, making treatments more effective and personalized, ultimately improving patient outcomes.

### 2.7 Hydrogels

Hydrogels are three-dimensional (3D) networks made up of hydrophilic polymer chains. With exceptional features, such as adjustable physical, chemical, and biological p0roperties, high biocompatibility, versatility in fabrication, and their resemblance to the native extracellular matrix (ECM), hydrogels have become promising materials in the field of biomedicine (Annabi et al., 2014). These polymeric networks can consist of natural polymers (chitosan, alginate, cellulose, starch, guar gum, collagens, proteins, acacia, and acid) or synthetic polymers (polyacrylic acid, polyacrylamide, polyvinyl pyrrolidone, acrylic acid, methacrylic acid, N-isopropylacrylamide, N-vinyl-2-pyrrolidone, etc.) soluble in water (Payal et al., 2021). The hydorgels have been used to treat some diseases, including cancer, cardiovascular diseases, and eye diseases. Recently, hydorgels has been explored in the aspect of treatment of dental caries.

Lori M. and coworkers developed Nitric oxide (NO)- and fluoride ion-releasing hydrogels with highly tunable biological properties suitable for combating pathogens at the root of dental caries infections. Nitric oxide (NO), a gaseous molecule produced endogenously, possesses broad-spectrum antimicrobial and antiviral capabilities, capable of penetrating and dispersing mature biofilms. It exterminates microbes by inflicting oxidative and nitrosative stress on lipids, proteins, metabolic transporters, and DNA (Nathan et al., 2018; Mark et al., 2021). The result through a 4 h viability study against *S. mutans* showed potent antimicrobial properties in eliciting a nearly 98% reduction in viable bacteria with the combination of GSNO and NaF gels, further *in vitro* testing of the fabricated gels against human osteoblasts and gingival fibroblasts demonstrated robust cytocompatibility over 4 and 24 hours. A further extended study showed combination gels exhibited reduced porosity after acid treatment, signifying the successful prevention of demineralization of the enamel-like substrates. These results encouraged further investigation of Hydrogels contain NO and fluoride to explore more promising ways in preventing dental caries (Lori et al., 2022).

Parmanand et al. developed selective small-molecule inhibitors which targets the *S. mutans*’ surface enzymes. And they have synthesized and demonstrated that the potent lead compounds HA5 was effective in the previous research. To enhance the solubility and antivirulence activities of the drug, they encapsulated HA5 within hydrogel microparticles, creating a hydrogel-encapsulated biofilm inhibitor (HEBI). The binding of HA5 to the glucosyltransferase GtfB was confirmed by resolving a high-resolution X-ray cocrystal structure of HA5 bound to the catalytic domain of GtfB and mapping its active site interactions. Additionally, HA5 effectively inhibited the glucosyltransferases of *S. mutans* and the production of glucan, with an IC50 value of 10.56μM in a Gtf inhibition assay. The HEBI demonstrated selective inhibition of the *S. mutans* biofilm similar to HA5. The result showed that treating *S. mutans* UA159-infected gnotobiotic rats with 100μM HA5 or HEBI for four weeks significantly reduced the scores for buccal, sulcal, and proximal dental caries compared to the control groups. This demonstrates their effectiveness in reducing virulence *in vivo* without significantly impacting bacterial colonization. During the test period, the rat had no loss of weight which demonstrated the compound HA5 or material HEBI have are nontoxic (Parmanand et al., 2023).

Qian et al. had produced amelogenin-derived peptide QP5, in their previous research, it can significantly promote the remineralization of the initial caries in the bovine enamel and the rat enamel without any toxic effects (Xuebin et al., 2015; Sili et al., 2017). The QP5’s application clinically is limited because its residence time with effective concentration on the tooth surface is relatively short when applied in the form of an aqueous solution. They added QP5 to chitosan hydrogel as a delivery system (CS-QP5 hydrogel) to evaluate the effects on *S. mutans* biofilm and test for its ability to promote remineralization. The result showed the *S. mutans* biofilm treated with the CS-QP5 hydrogel had a lower CFU count, lactic acid production, and metabolic activity compared with the other groups even after seven days. That’s probably because a sustainable retention of the hydrogel on the tooth surface provided a consistent number of effective agents over a prolonged period of time, which may decrease the cariogenic property of the dental biofilm with fewer potential side effects. In polarized light microscopy, after inoculation for 1 day, a surface layer was clearly visible on the enamel treated with the CS-QP5 hydrogel. With an increase in the inoculation time, the lesions became significantly shallower and the negative birefringence of the enamel surface layer became more obvious in the CS-QP5 hydrogel group than in the other four groups. In addition, the CS-QP5 hydrogel group showed a significantly higher mineral content than the other groups on each day, these results provided direct evidence of the remineralization promoted by the CS-QP5 hydrogel. Therefore, as a promising deliver system of active substances, hydrogel has been proven to increase antibacterial ability which is promising application in treatment (Qian et al., 2018).

Hydrogels have emerged as promising biomaterials in biomedicine, with increasing interest in their applications for dental caries treatment. Innovations like hydrogels containing nitric oxide and fluoride ions provide novel strategies against dental caries pathogens. Additionally, QP5-enriched hydrogels demonstrate efficacy in remineralization and biofilm reduction, highlighting their potential in caries management. Future research should optimize these formulations for improved retention, effectiveness, and safety.

### 2.8 Metal nanoparticles

#### 2.8.1 Silver nanoparticles

Silver ions are widely used because of their low toxicity, broad-spectrum antimicrobial characteristics, and the lack of cross-spectrum bacterial resistance (Claudia Butrón-Téllez et al., 2020). Compared to ordinary silver ions, nanosilver further increases the relative surface area, and its ability to easily penetrate biological and structural barriers results in better antimicrobial effects (Ammar, 2022). Silver nanoparticles (AgNPs) exhibit exceptionally potent toxicity toward microbes, boasting antibacterial properties 25 times stronger than those of CHX. They also possess antiviral, antifungal (Besinis et al., 2014), and anti-tumor cell activities (Noronha et al., 2017). Consequently, numerous studies have recommended their integration into various formulations, where they have demonstrated effective results in the prevention and treatment of early dental caries.

As carriers of transporting drug molecules, AgNPs can reduce side effects and enhance therapeutic effects. In addition, the use of silver nanoparticles in conjunction with other antibiotics has a stronger antibacterial effect. Studies have reported that the combined use of AgNPs with chlorhexidine and metronidazole exhibits effective bacteriostatic and bactericidal properties (Nikita et al., 2019). However, before this, there were no reports of attaching chlorhexidine or metronidazole to the surface of AgNPs. To explore the possibility of using AgNPs as carriers for drugs such as chlorhexidine and metronidazole in treating oral bacterial infections like periodontitis and other diseases. Karol P. Steckiewicz et al. developed a new type of silver nanoparticles, which are combined with Chlorhexidine (AgNPs-CHL) and Metronidazole (AgNPs-PEG-MET) (Karol et al., 2022). These innovative compounds, whether it’s AgNPs-CHL or AgNPs-PEG-MET, exhibit potent antibacterial, anti-biofilm, and anti-inflammatory properties. *In vitro* models assessing the safety of its potential clinical applications have demonstrated that these silver nanoparticles (AgNPs) exhibit low cytotoxicity at high concentrations; at non-toxic concentrations, they are harmless to mammalian cells. Importantly, findings suggest that AgNPs serve as effective drug delivery carriers for Chlorhexidine and Metronidazole. However, the exploration of AgNPs as carriers for Chlorhexidine or Metronidazole in the prevention and treatment of dental caries remains uncharted territory, presenting an exciting opportunity. The anticipation is high for future researchers to delve into this area, potentially paving the way for using silver nanoparticles as drug carriers in the fight against dental caries.

Besides serving as carriers, AgNPs also demonstrate potent antibacterial effects. AgNPs adhere to the cell wall and cytoplasmic membrane through electrostatic attraction and affinity with thiol proteins, and they can change the permeability and structure of bacterial cells. When AgNPs enter the cells, they inhibit respiratory enzymes, thereby generating reactive oxygen species, causing oxidative stress response, while also interfering with DNA and inhibiting protein synthesis, leading to changes in cell structure and cell death (Lakshmi et al., 2021). Research has also found that AgNPs can inhibit *S. mutans* and its biofilm, thus having a wide range of clinical applications in the prevention of dental caries (Mario Alberto et al., 2015), for example, adding silver nanoparticles to orthodontic brackets has satisfactory effects in inhibiting *S. mutans* and preventing enamel surface caries (Gamze et al., 2016).

#### 2.8.2 Zinc nanoparticles and zinc oxide nanoparticles

In recent years, zinc nanoparticles have shown promising anti-tumor (Sumaira et al., 2021), antibacterial, and antiviral properties (Mahda Sadat et al., 2022), with their oxides, ZnONPs, demonstrating superiorities. Owing to their expanded specific surface area, potent antibacterial activity, and excellent biocompatibility, ZnONPs demonstrate significant benefits and are therefore extensively employed in a variety of applications, including dental implants, prosthetic joints, and cardiovascular implants (An overview on zinc-oxide nanoparticles as novel drug delivery system, 2023). In the field of drug delivery, ZnONPs are considered potential candidates for targeted drug delivery carriers. Literature has reported on ZnONPs coupled with arginine-glycine-aspartic acid (RGD) peptides, as well as ZnONPs loaded in the form of a metal-doxorubicin (DOX) complex, for targeted cancer therapy (Xiaoxin et al., 2019). However, no literature was retrieved on ZnONPs as drug carriers targeting the prevention and treatment of dental caries, so only the role of ZnONPs as nano-drugs in inhibiting oral cariogenic bacteria and biofilms is introduced below. The mechanisms by which ZnONPs inhibit bacteria include the following: (1) Through the cell’s oxidative stress response, harmful oxidizing compounds, specifically H_2_O_2_, are generated inside the cell body to exert activity. As the concentration of zinc oxide nanoparticles increases, the production of H_2_O_2_ increases, and the bactericidal effect is correspondingly enhanced. (2) ZnONPs counteract bacteria by releasing Zn^2+^ ions, and Zn^2+^ ions can destroy cell membranes and internal components of the cell. (3) The presence of negative charges on the surface of microorganisms, while metal oxides carry a positive charge, leading to electromagnetic attraction between microbes and zinc nanoparticles. If attraction occurs, the microorganisms are oxidized and die rapidly (Tahreem et al., 2022). One reason for the inhibition of *S. mutans* by ZnONPs is that ZnONPs can inhibit the biofilm of dental plaque, penetrate the biofilm, disperse with the matrix, both generate reactive oxygen species (ROS) and destroy membrane transport. Therefore, Hamad et al. explored the direct effects of ZnONPs on *S. mutans*, finding that they have high antibacterial activity against both Gram-positive and Gram-negative bacteria, and the excellent antibacterial activity of ZnONPs increases with their concentration (Fairoz Ali et al., 2022). In a series of clinical experiments, for example, zinc oxide nanoparticles incorporated into dental restorations can be used to control and prevent secondary caries (Arshad Mahdi and Qanat Mahmood, 2021). Malekhoseini et al. developed a resin-modified glass ion polymer containing ZnONPs to enhance its physical properties and antibacterial potential (Zahra et al., 2021). It has been reported that the addition of ZnONPs into composite resins found that 1% ZnNPs had no significant effect on the mechanical properties of the composite resin, and as the mass fraction increased to 5%, the number of *S. mutans* significantly decreased within one day (Zahra et al., 2021). These experiments suggest that zinc and its oxide nanoparticles can be used independently or in conjunction with other antimicrobial agents in resins or adhesives, and nanoparticles may gradually emerge as significant in future bonding or filling materials.

#### 2.8.3 Titanium nanoparticles

Due to the high ratio of surface area to volume and high reactivity, nanoparticles have become effective antimicrobial agents. Among these, titanium nanoparticles are extensively utilized in the medical field owing to their excellent reflectivity, chemical stability, biocompatibility, bioactivity, and broad antimicrobial activity (Amir Hossein et al., 2021). In the field of dentistry, titanium dioxide nanoparticles are one of the titanium nanoparticles that have been extensively studied for caries prevention. Mohammed et al. incorporated TiO_2_NPs into resin composites and adhesives to assess whether the addition of TiO_2_NPs would enhance the antimicrobial activity of the composite materials (Wongchai et al., 2023). One month after the experiment started, plaque was collected from the gingival margin to determine the number of *S. mutans* in the plaque. It was found that the surface of restorations with added TiO_2_NPs showed a significant reduction in plaque, especially when TiO_2_NPs were added to both resin composites and adhesives. More notably, the antimicrobial activity increased over time. Additionally, incorporating antimicrobial agents such as silver and TiO_2_NPs into orthodontic adhesives enhanced the antimicrobial performance against *S. mutans* and *Lactobacillus acidophilus* compared to commercially available fluoride-containing composites (Mohammed et al., 2018). Therefore, adding TiO_2_NPs to dental restorative materials can strengthen their anti-caries effect. The combined use with other metal nanoparticles may achieve even more ideal antimicrobial effects. These nanoparticles may be used in the development of anti-caries restorative materials in the future. However, before this, adequate *in vivo* experiments are needed to evaluate the potential side effects of these metal nanoparticles and further optimize their physicochemical properties and biosafety.

### 2.9 Cell membrane-coated nanoparticle (CMCNP) technology combined with nanodrug delivery systems

Recently, cell membrane-coated nanoparticles (CMCNPs) have attracted widespread attention as a novel nanodelivery system. This technique involves encapsulating nanoparticles with cell membranes, almost completely preserving the complexity of the cell membrane on the surface of the particles. Therefore, CMCNPs not only possess the advantages of nanoparticles but also retain numerous functions of the source cell membrane, such as the ability to interact with other cells. Due to the unique physiological structure of cariogenic biofilms, the effectiveness of traditional nanosystems for drug delivery is limited, this method utilizes the natural functions of the cell membrane, allowing nanoparticles to demonstrate great potential in the diagnosis and treatment of various diseases (Mahendra et al., 2022). Members of our research group, Weng et al. inspired by cell coating technology and combining the ability of *Lactobacillus strains* to adhere to *S. mutans*, coated *Lactobacillus* (LA) cell membranes onto PLGA nanoparticles carrying Triclosan (TCS) (LA/TCS @ PLGA-NPs) (Zou et al., 2020). We discovered that this composite nanoparticle demonstrated good encapsulation, controllable size, negative charge, and sustained-release kinetics inheriting the natural characteristics of the original cell surface. As a result, LA/TCS @ PLGA-NPs could adhere to *S. mutans* and extend the drug release time, thereby continuously inhibiting the formation of *S. mutans* biofilm and having a lasting inhibitory effect on the progression of dental caries. Similarly, members of our subject matter team Ye et al. constructed a biomimetic oral mucosal adhesive drug delivery system, employing *Streptococcus salivarius* K12 membrane-coated TCS @ PLGA-NPs (Lu-Ting et al., 2022; Jiajia et al., 2023). This composite could adhere to the oral mucosal epithelium and promote the antibacterial action of TCS @ PLGA-NPs at the infection site, while the outer membrane of *Streptococcus salivarius* acted as a diffusion barrier for TCS release, extending the duration of drug action. These two studies by our research group, which combined cell coating technology with nanosystems for drug delivery, improved the precision of targeted drug delivery, facilitating more effective binding to the infection site and optimizing drug delivery, thereby boosting therapeutic effects. It offers inspiration for combining nanodelivery systems with other technologies, presenting novel strategies for the future by inhibiting the formation of plaque biofilms or suppressing bacterial growth to prevent and treat dental caries (**Table 1**).

**Table 1:** The studies that used nanodrug delivery systems for caries prevention and treatment.

Ways of Anti-caries	Nanosystems for drug delivery	Types of drug carried or Combination	Response	Modify Materials	Mechanism	conclusion	Model In Vivo/Vitro Experiment	Ref
	Liposomes	Mag and FLC PPI、histatin-1、indocyanine green	Good affinity for Hydroxyapatite、Higher surface microhardness recovery	\	(1) Deliver and protect the drug	nano-encapsulated lactoferrin reduced the CFU of biofilm up to two logarithmi cycles.	Biofilm、synthetic and biological hydroxyapatite	Luo et al., 2023; Wang et al., 2018a; Wang et al., 2018b; Xiao et al., 2023
					(2) Fuse into the cell surface and release drug			
	poly-(lactic-co-glycolic acid) (PLGA)	Chlorhexidine、Chlorhexidine diacetate (CDA) and digluconate (CDG)	\	adhesive	protect drug from degradation and provide a sustained drug release profile	TEM confirmed successful tubular penetration and retention of Nano-PLGA/CHX nanoparticles inside the structure of dentinal-tubules.	micron-sizeddentinal-tubules under pulpal-pressure simulated	Priyadarshini et al., 2017; Francisco Fábio Oliveira de et al., 2021
	Mesoporous silica nanoparticles	L-arginine、Metal particle	\	adhesive	Being able to deliver L-arginine in a sustained way with a long-term antibacterial activity enough to selectively inhibit the growth of cariogenic bacteria such as *S. mutans* and *L. casei*	relative light units(RLUs) of *S. mutans* reduce	Biofilm of *S. mutans* and *L. casei*	Marta et al., 2022; Lu et al., 2018; Yuting et al., 2022?;
	poly(aminoamine) (PAMAM)	Honokiol、NGV peptides and galardin、	\	adhesive	Drug can be encapsulated within the PAMAM dendrimer or conjugated to surface groups	triclosan encapsulated in the formulation are able to release in a controlled manner	Artificial Saliva、Dentin disks	Tao et al., 2021; Tao et al., 2022; Menglin et al., 2020
	Chitosan	FNA、Amoxicillin、Cinnamaldehyde	\	\	As the carrier of drug with biocompatibilitybiodegradability and bioadhesivity	higher fluoride uptake ability and smooth releasing profile	In vitro characterization of nanoparticles	Jiang et al., 2024; Ran et al., 2023
Anti-biofilm	Polymeric micelles	tannic acid、NaF、DpSpSEEK	PH-sensitive	\	Solubilize drug and enhance its bioavailability	exerts effective antibacterial adhesion and cariogenic biofilm resistance	Both in vitro and in vivo	Xu et al., 2023
	silver nanoparticles	\		adhesive 、resin		Having Stronger inhibit caries and better in the dental remineralization ability	decayed	Karol et al., 2022
				Prosthetics-materials、commercial fluoride varnish、a pit and fissure sealant、			teeth in vivo	
					(1)increasing the absorbency of membrane			
	Zinc oxide nanoparticles	\		Toothpastes、resin、adhesive、sealer	(2)Destroy the DNA, proteins, and lipids are among the bacterial components	zinc oxide have an excellent anti-bacterial benefit and it can be better by increasing the solution concentration that Contains the nanoparticles.	The transferred Staphylococcus and Streptococcus in Mannitol salt agar and MSB agar base	Tahreem et al., 2022; Fairoz Ali et al., 2022
			Oxidative stress		(3)cause bacterial cell death by inducing an oxidative stress response			
	Titanium Nanoparticles	\		adhesive 、resin		TiO2NPs enhanced the antibacterial activity	Teeth in vivo	Wongchai et al., 2023; Amir Hossein et al., 2021
	Nano-Hydroxyapatite	\	\	fluoride varnish、Toothpastes	nHA remineralizes the organic scaffold in the carious attack by directly replacing lost minerals or as a carrier for lost mineral ions and increasing the supply of calcium and phosphorus ions	Remineralising effects of NanoHAP dentifrice were found to be significantly superior to routine fluoridated dentifrice.	Teeth of therapeutic extraction for the orthodontic treatment in vivo	Verma and Muthuswamy Pandian, 2021; Juntavee et al., 2021
	Nano-sizedamorphous calciumphosphate Particle	Dimethylaminohexadecyl methacrylate (DMAHDM)	\	adhesive	Nanoparticles of amorphous calcium phosphate (NACP), able to discharge much more Ca and P by virtue of the tiny particle size and large surface area	NACP+DMAHDM release a great deal of Calcium and phosphate had the highest microhardness compared NACP+DMAHDM group	Artificial initial carious lesion in Bovine incisors	Fan et al., 2022
Remineralization								
	nano-sized calcium fluoride	DMAHDM	\	experimental resins monomers	The smaller particle size and higher surface area achieved with the use of nCaF2 enabled a higher level of F ion release	nCaF2+DMAHDM composite showed a significant fluoride ion concentration	dental plaque microcosm biofilm	Daixing et al., 2022
	Bioactive Glass Nanoparticles	Ag	\	\	Antibacterial:BG release ionic dissolution products leading to the damaging of bacterial cell wall by BG sharp debris	XRD characterization: hydroxy-carbonate apatite layer formation on their surfaces following the immersion in SBF	simulated body fluid	Kazemian et al., 2021
					Remineralization:Ca2+ and P2+ can combine in solution and deposit onto silanol bonds on the glass surface, nucleating a hydroxycarbonate apatite layer.			

## 3 Remineralization

### 3.1 Nano - hydroxyapatite (nHAp)

Hydroxyapatite constitutes the main inorganic component of dental hard tissues. With the emergence of nanotechnology, the application of nHAp has become increasingly widespread due to its excellent mechanical, physical, and chemical properties. The nHAp has higher solubility and surface energy as well as better biocompatibility, and the morphology and structure of nHAp particles are similar to the hydroxyapatite crystals in teeth (Pushpalatha et al., 2023). Furthermore, as a biocompatible synthetic material, it can serve as a source of Ca^2+^ and PO_4_^3-^ in the oral cavity, particularly under acidic conditions. Increasing the levels of Ca^2+^ and PO_4_^3-^ can significantly limit the enamel damage caused by acids, thereby substantially enhancing the degree of remineralization (Aiswarya et al., 2022), with the effect of mitigating demineralization and fostering remineralization, with the effect of mitigating demineralization and fostering remineralization. The nHAp chemically combines with natural hydroxyapatite crystals to form a uniform hydroxyapatite layer on the surface of demineralized enamel, thus inducing remineralization. The hardness and elastic modulus of the repaired enamel are similar to those of natural enamel. Additionally, literature indicates that nHAp, due to its high surface energy, can strongly bind to the enamel surface and fill the gaps and micropores on the enamel surface to repair it (Nebu, 2018; Aref and Alsdrani, 2023). The literature indicates that nHAp can repair early enamel damage and has the potential to serve as an auxiliary repair material as well as prevent acid erosion damage, making it a safe and effective anticaries agent in oral care (Paszynska et al., 2023). Apa Juntavee first compared the effects of two concentrations of nanohydroxyapatite gel (NHG, 20% and 30%) with nHAp -containing toothpaste (NHT) and fluoride varnish (FV) in the remineralization of artificial carious lesions (Juntavee et al., 2021). Throughout the remineralization process, the microhardness of both the tooth surface and cross-sections was evaluated, and the depth of the caries was analyzed through polarized light microscopy photos. The research results indicate that the remineralization effect of nHAp toothpaste is superior to that of two types of nanohydroxyapatite gels, and the effects of both products containing nHAp exceed those of fluoride varnish. This also demonstrates that, whether in the form of toothpaste or gel, nanohydroxyapatite’s ability to remineralize is better than fluoride.

To explore the effectiveness of toothpaste containing nHAp on a model of demineralized teeth within the body, Purva Verma et al. treated teeth that had undergone orthodontic bracket detachment (due to changes in surface demineralization) separately with fluoride toothpaste and nHAp toothpaste for 15 days (Verma and Muthuswamy Pandian, 2021). Subsequently, Atomic Force Microscopy was employed to analyze the surface roughness, which served as a measure to evaluate the remineralization potential of the toothpaste. It showed the hydroxyapatite nanoparticles are incorporated into oral care products to facilitate enamel remineralization through ion supersaturation at the site of lesions, a mechanism analogous to that of other calcium-based nanoparticles. Nonetheless, these nanoparticles enhance enamel regeneration by generating a biomimetic film that resembles biological hydroxyapatite, demonstrating greater efficacy in caries repair *in vitro* when compared to fluoride and casein nanoparticles. This phenomenon occurs because the remineralization layer exhibits resistance to abrasion due to the chemical bonds formed between the synthetic and natural crystals of the enamel, even in a potentially cariogenic environment characterized by a pH of 4.

In the future, as nanotechnology advances, the application of nHAP may expand to a broader range of caries prevention, remineralization, and restorative materials, making it an essential component of oral health maintenance. The incorporation of nHAP in oral care products is expected not only to enhance product performance but also to influence consumer choices regarding oral health products, driving the development of dental materials toward safer and more effective alternatives.

### 3.2 Nano-sized amorphous calciumphosphate Particle (NACP)

Calcium phosphate (CaP) is a substance containing Ca^2+^ and PO_4_^3-^, Amorphous calcium phosphate (ACP) is an intermediate phase formed during the precipitation process of CaP, and the natural solid form of ACP is usually composed of a group of ACP nanoparticles, with a specific surface area of about 300m^2^/g (Sun et al., 2019). Therefore, materials containing ACP nanoparticles are highly regarded for their effect in promoting remineralization. In a comparative study conducted by Tao et al. on the impact of NACP-containing adhesives versus commercially available fluoride-releasing adhesives on dentin remineralization in a biofilm setting, it was observed that NACP exhibited bacterial inhibition in a simulated oral environment (Tao et al., 2019). Adhesives containing NACP, by releasing Ca^2+^ and PO_4_^3-^, contribute to tooth mineralization. A measurement of the Ca^2+^ and PO_4_^3-^ content in the biofilm after 10 days indicated that the adhesive with NACP, through the release of these ions, not only increases the concentration of Ca^2+^ and PO_4_^3-^ in the biofilm but also restores the hardness of the dentin and achieves the sealing of the dentinal tubules, thereby confirming its effectiveness in dentin remineralization. In contrast, commercial fluoride-releasing adhesives merely inhibit further demineralization. Similarly, Fan et al. evaluated the enamel remineralization effect in the oral biofilm environment by combining antimicrobial agents and remineralizers (Fan et al., 2022). They used NACP as the remineralizer and found that the PO_4_^3-^ in NACP could react with H^+^ in the biofilm, thereby stopping further pH value decrease. Adhesives containing NACP were able to effectively remineralize hard dental tissues, protect the adhesive interface, and inhibit secondary caries. Moreover, the study found that the release of Ca^2+^ and PO_4_^3-^ from NACP alone might offer better localized acid production limiting capabilities than using the antimicrobial agent Dimethylaminohexadecyl methacrylate (DMAHDM) alone. These findings suggest that adding NACP to dental repair materials could effectively promote enamel adhesion, protect the adhesive interface, prevent caries, and extend the lifespan of composite materials, showing broad application prospects.

### 3.3 Nano-sized calcium fluoride (NCaF2)

NCaF2 has been proven to effectively prevent dental caries by increasing the fluoride concentration in oral saliva, which promotes the remineralization of teeth (Sun and Chow, 2008). Additionally, it can be used to reduce the permeability of dentin. Heba Mitwalli developed a novel composite material containing dimethylaminohexadecyl methacrylate (DMAHDM) and calcium fluoride nanoparticles (NCaF2), aimed at preventing the occurrence of secondary caries at the margins of restorations (Heba et al., 2020). This material inhibits bacterial growth through the antibacterial properties of DMAHDM and significantly releases fluoride and calcium ions through the inclusion of NCaF2. Fluoride ions, acting as transmembrane proton carriers and inhibitors of glycolytic enzymes, can stimulate cytoplasmic acidification, exerting antibacterial effects at a distance and thereby preventing the formation of caries (Daixing et al., 2022). Additionally, fluoride ions can also promote the formation of fluorapatite, enhancing the remineralization effect on teeth. This novel composite exhibits strong antibacterial and ion-release capabilities, preventing the continued production of lactic acid by biofilms and significantly reducing the number of *S. mutans* on the biofilm. This bioactive nanocomposite material holds promise as a new type of anticariogenic material that protects tooth structure, inhibits demineralization, and serves as a reservoir for the release of calcium and fluoride ions.

### 3.4 Bioactive glass nanoparticles (BGN)

Bioactive glass (BAG) is highly valued as a regenerative material due to its controllable degradability and ability to stimulate tissue formation. It can promote bone induction and thereby bone formation by releasing ionic products. With the combination of tissue engineering and nanoscience, nanomaterials capable of imitating the characteristics of host tissues, such as bioactive glass nanoparticles (BGN), have emerged. These can adjust their size according to the host’s response to facilitate cellular absorption, allowing therapeutic ions to be released inside cells (Shalumon et al., 2013; Jones, 2015; Pajares-Chamorro and Chatzistavrou, 2020).In the field of dentistry, BGN, due to its controlled ion release capability, has been applied to the remineralization of enamel and dentin (Bakry et al., 2014). It composed of sodium-calcium-amorphous phosphate, reacts with H^+^ upon contact with saliva, releasing Ca^2+^ and PO_4_^3-^, leading to an immediate increase in pH value, which promotes the deposition of calcium and phosphate, followed by the formation of a hydroxyapatite layer (Taha et al., 2017). Compared to BAG, BGN has a higher ion release capacity and bioactivity, therefore, it has a wide range of application prospects in the aspect of tooth remineralization.

Zahra Kazemian et al. have prepared BGN containing silver ions using the sol-gel method, which can change the pH value of the surrounding medium through ion release (Kazemian et al., 2021). The dissolution of BG leads to the supersaturation of calcium ions in simulated body fluid, and results in the formation of reprecipitation on its surface. These precipitates, rich in calcium and phosphorus crystals, have been verified by X-ray diffraction, confirming that the hydroxyapatite layer formed on the surface of Ag-BG after soaking in simulated body fluid. Furthermore, Akbarzadeh et al. synthesized a paste containing nano-sized bioactive glass (BGN) amorphous calcium phosphate casein phosphopeptide (CPP-ACP) paste, and compared its remineralization capability with that of fluoride-containing CPP-ACP paste and commercial products (Akbarzade et al., 2022). Observations through Vickers microhardness test and scanning electron microscope (SEM) revealed that both pastes could promote the remineralization of demineralized dental enamel. However, the enamel treated with the paste containing BGN showed stronger microhardness compared to that treated with the fluoride-containing CPP-ACP paste and commercial products. Although the remineralization effect of BGN is initially recognized, the potential of different types of bioactive glass (BGs) for remineralization *in vitro* and *in vivo* still requires further research.

In addition to Bioactive Glass Nanoparticles, Mesoporous Bioactive Nanoparticles (MBN) also receive considerable attention in the field of nanomedicine due to their ability to load and deliver biomolecules, along with their nanoscale and bioactive component features. For instance, in the study conducted by Jung-Hwan Lee et al., they developed a novel drug delivery system that utilizes BGN to load strontium ions (Sr^2+^) and phenamil (Lee et al., 2017). Sr^2+^ can promote the differentiation of precursor cells or stem cells into osteogenic cells/cementoblast thereby stimulating the growth of these cells (Kruppke et al., 2019; Miyano et al., 2023).Sr^2+^ can interact with intracellular signaling molecules in the body, and they stimulate the repair of hard tissues through the BMP2/Smad signaling pathway; similarly, phenamil has also been proven to be a potent small molecule BMP signaling activator, which enhances BMP2/Smad when acting on osteoblasts cell or cementoblast (Park et al., 2009; Xu et al., 2020). During the experimental process, they observed the formation of poorly crystallized hydroxyapatite phase in the composite (Sr^2+^/phenamil—MBN) on the third day of soaking in simulated body fluid, and the density increased with the soaking time. *In vivo* studies, they created a drill hole defect on the extracted tooth surface, filled the defect with Sr/phenamil MBN to make it contact with dental pulp tissue as much as possible and interact with mesenchymal stem cells (MSCs), and implanted it into the subcutaneous sites of rats. Six weeks later, three-dimensional constructions and two-dimensional projection images obtained by micro-CT revealed new hard tissue formation in the tooth drill hole defect area, and histological analysis also confirmed that the new tissue was bone or dentin. They achieved for the first time the co-delivery of ions and drug molecules on nanoparticles and successfully facilitated hard tissue regeneration, indicating that MBN loaded with strontium ions and phenamil is a highly potential drug delivery platform. BGN offers broad applications in tooth remineralization due to its high ion release and bioactivity, though further research is needed on various bioactive glasses (BGs). Mesoporous Bioactive Nanoparticles (MBNs) also attract attention for drug delivery. The co-delivery method provides a new strategy for caries remineralization, suggesting innovative approaches in dental care.

## 4 Conclusion

In recent years, nanotechnology has increasingly attracted attention in the field of dentistry. The development of this technology has made it possible to prevent and treat oral diseases, especially dental caries, by designing and creating nanoscale drug delivery systems. These systems can directly deliver drugs to the lesion sites of teeth, effectively preventing and treating dental caries. Moreover, studies have shown that nanoscale drug delivery systems enhance the therapeutic effect of drugs by increasing their bioavailability, thus reducing the side effects of drugs to strengthen the treatment effect. Meanwhile, researchers are also exploring the remineralization potential of nanoparticles, intending to help repair teeth damaged by dental caries by mimicking the natural mineralization process of teeth.

Future research in nanomedicine for targeted therapy aims to achieve precise drug delivery to biofilm-infected sites using diverse nanoparticles, while safeguarding the physiological function of healthy cells. This encompasses the development of adaptive drug release mechanisms responsive to physiological changes. Concurrent efforts will prioritize the optimization of nanocarriers, exploring advanced biomaterials and stimuli-responsive nanomaterials, integrating functionalities such as drug delivery, therapeutics, and diagnostics. Crucially, expediting clinical translation is essential, involving scalable manufacturing, comprehensive safety evaluations, and the establishment of clear regulatory frameworks. Novel therapeutic paradigms, including the refinement of immunotherapies, gene therapy, RNAi therapies, and the development of antimicrobial agents combating resistance, will significantly benefit from nanotechnological advancements. Furthermore, the evolution of nanomedicine in medical imaging and diagnostics, specifically via highly sensitive imaging agents and disease biomarker sensors, promises to be transformative. Lastly, employing artificial intelligence and machine learning in nanomedicine design, data analysis, and virtual screening will accelerate the entire research and development process.

Despite notable progress in dental applications of nanotechnology, several pivotal research gaps remain. Currently, the field lacks standardized evaluation protocols to systematically assess the safety and efficacy of nanosystems for oral use, resulting in limited cross-study comparability. Establishing uniform standards is essential for accurately comparing and evaluating disparate materials and approaches. Furthermore, while some nanomaterials exhibit promising laboratory and small-scale trial results, their transition to clinical practice is hampered by various challenges. Comprehensive *in vivo* studies and larger clinical trials are crucial to refining these materials’ physicochemical properties and confirming their biocompatibility. With robust clinical evidence, nanosystems can become integral to standard dental treatments, providing reliable solutions. The interaction between nanosystems and the oral microbiome is another under-researched domain. The oral microbial community’s complexity means that nanomaterials may engage in intricate interactions with both beneficial and harmful bacteria, influencing ecological stability and balance. In-depth investigations into these dynamics are necessary for designing nanotechnologies that effectively regulate microbial communities while minimizing adverse effects. Lastly, advancements in biomaterials and nanotechnology have ushered in emerging trends such as smart drug delivery systems and biomimetic materials, offering prospects for personalized and adaptive therapies. Although in nascent stages, these innovative technologies hold the potential to revolutionize dental care paradigms. Continued basic research alongside applied development will be pivotal in advancing these transformative applications.
